# Contribution of mitochondrial gene variants in diabetes and diabetic kidney disease

**DOI:** 10.3389/fendo.2022.953631

**Published:** 2022-10-12

**Authors:** Meng Li, Siqian Gong, Xueyao Han, Lingli Zhou, Simin Zhang, Qian Ren, Xiaoling Cai, Yingying Luo, Wei Liu, Yu Zhu, Xianghai Zhou, Yufeng Li, Linong Ji

**Affiliations:** ^1^ Department of Endocrinology and Metabolism, Peking University People’s Hospital, Peking University Diabetes Center, Beijing, China; ^2^ Department of Endocrinology, Pinggu Teaching Hospital, Capital Medical University, Beijing, China

**Keywords:** mitochondrial DNA, mitochondrial diabetes, diabetes, diabetic kidney disease, insulin resistance

## Abstract

**Objectives:**

Mitochondrial DNA (mtDNA) plays an important role in the pathogenesis of diabetes. Variants in mtDNA have been reported in diabetes, but studies on the whole mtDNA variants were limited. Our study aims to explore the association of whole mtDNA variants with diabetes and diabetic kidney disease (DKD).

**Methods:**

The whole mitochondrial genome was screened by next-generation sequencing in cohort 1 consisting of 50 early-onset diabetes (EOD) patients with a maternally inherited diabetes (MID) family history. A total of 42 variants possibly associated with mitochondrial diseases were identified according to the filtering strategy. These variants were sequenced in cohort 2 consisting of 90 EOD patients with MID. The association between the clinical phenotype and these variants was analyzed. Then, these variants were genotyped in cohort 3 consisting of 1,571 type 2 diabetes mellitus patients and 496 subjects with normal glucose tolerance (NGT) to analyze the association between variants with diabetes and DKD.

**Results:**

Patients with variants in the non-coding region had a higher percentage of obesity and levels of fasting insulin (62.1% vs. 24.6%, P = 0.001; 80.0% vs. 26.5% P < 0.001). The patients with the variants in rRNA had a higher prevalence of obesity (71.4% vs. 30.3%, P = 0.007), and the patients with the variants in mitochondrial complex I had a higher percentage of the upper tertile of FINS (64.3% vs. 34.3%, P = 0.049). Among 20 homogeneous variants successfully captured, two known variants (m.A3943G, m.A10005G) associated with other mitochondrial diseases were only in the diabetic group, but not in the NGT group, which perhaps indicated its possible association with diabetes. The prevalence of DKD was significantly higher in the group with the 20 variants than those without these variants (18.7% vs. 14.6%, P = 0.049) in the participants with diabetes of cohort 3.

**Conclusion:**

MtDNA variants are associated with MID and DKD, and our findings advance our understanding of mtDNA in diabetes and DKD. It will have important implications for the individual therapy of mitochondrial diabetes.

## 1 Introduction

Mitochondrial diseases are a clinically heterogeneous cluster of inherited metabolic disease that could affect the function of the mitochondrial respiratory chain and oxidative phosphorylation system (1). Diabetes is the most well-described form of endocrine dysfunction in mitochondrial diseases. The mitochondrial genome is a maternally inherited 16,569-bp circular double-stranded DNA molecule that contains two ribosomal RNA genes, 22 transfer RNA (tRNA) genes, and 13 genes for the proteins of the oxidative phosphorylation system (2). Mitochondria are important for the synthesis of ATP and synthesis of the hormone. The mitochondrial DNA (mtDNA) variants possibly lead to the lack of ATP and impair hormone production when mitochondrial dysfunction occurs. In the process of insulin synthesis, mitochondria not only provide the necessary ATP for insulin exocytosis but also have a core function in glucose sensing and the induction of triggering and amplifying signals that adjust insulin secretion to glycaemia. Variants in the mitochondrial genomes are related to a cluster of metabolic diseases, including mitochondrial diabetes, insulin resistance (IR), non-alcoholic fatty liver disease (NAFLD), and metabolic syndrome (MetS) (3–6). Single mtDNA variants are associated with type 2 diabetes mellitus (T2DM). An mtDNA genome-wide association analysis indicated a potential role for mtDNA in diabetic kidney disease (DKD). As we know, the m.A3243G variant is the most common heteroplasmic mtDNA variant associated with mitochondrial diabetes. The m.A3243G variant reduced the synthesis of mtDNA-encoded proteins, which resulted in an imbalance within the mitochondrion between nuclear and mitochondrial encoded oxidative phosphorylation subunits (7, 8). Multisystemic disease manifestations can precede the onset of diabetes in patients with mitochondrial diseases (9). For example, sensorineural deafness and a family history of maternal inheritance should raise suspicion that the patient might have a mitochondrial disease. Other mtDNA variants associated with diabetes include point variants in the tRNA genes MT−TL1, MT−TK, MT−TS2, and MT−TE, as well as the variants in MT−ND6, which encodes the NADH-ubiquinone oxidoreductase chain 6 (10–12). Furthermore, IR also plays a key role in the development of T2DM and DKD. There is a clear evidence of an association between skeletal muscle IR and abnormalities in mitochondrial oxidative metabolism (13). However, most studies just focused on the association among single mtDNA loci and diabetes and DKD, and studies on the whole mtDNA gene with diabetes and DKD were limited. In addition, high-throughput sequencing could enhance the ability to identify mitochondrial respiratory chain complex deficiencies (14). Therefore, the study was designed as follows: to screen the whole mitochondrial gene variants in early-onset diabetes with the maternal family history using next-generation sequencing (NGS) and then to explore the mtDNA variants with diabetes and DKD. The study aims to help us elucidate novel insights into mitochondrial genetic etiology.

## 2 Materials and methods

### 2.1 Study overview

**Figure 1:**
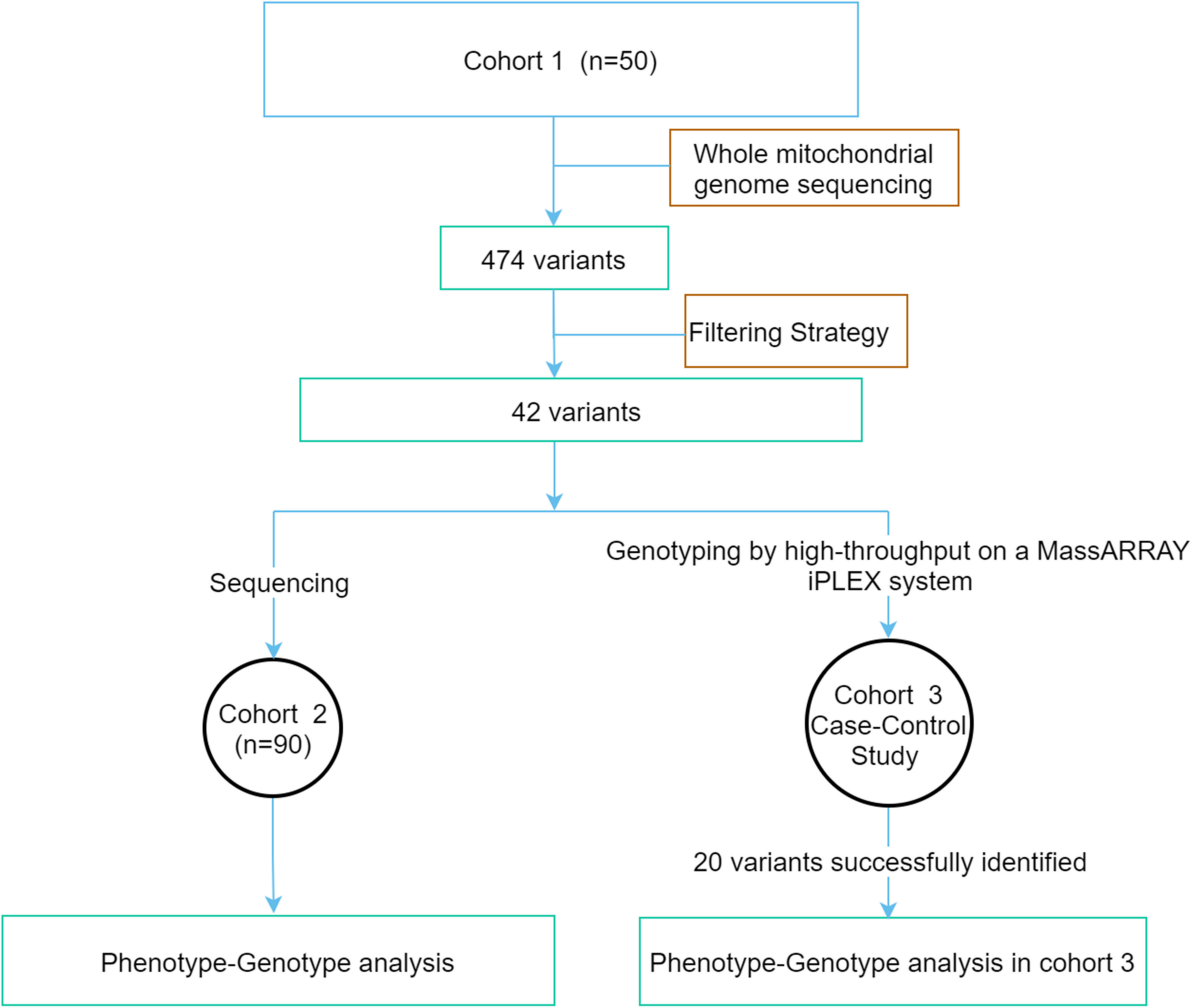
The flowchart of the study.

This study was conducted in compliance with the Declaration of Helsinki. The study protocol was approved by the Ethics Committee of Peking University People’s Hospital. Written informed consent was obtained from all the study participants. A flowchart of the study is shown in **Figure 1**. The study was divided into three groups (1): The whole mitochondrial genome was screened by NGS in cohort 1 consisting of 50 early-onset diabetes (EOD) patients. The variants possibly associated with mitochondrial diseases were identified according to the filtering strategy. (2) The above variants were sequenced in cohort 2 with 90 EOD patients, and the clinical phenotypic features of patients with potential pathogenic variants were analyzed. (3) Cohort 3 was a case–control study that included 1,571 patients with T2DM and 496 healthy individuals with normal glucose tolerance (NGT) to analyze the different features of the patients with variants and without these variants.

### 2.2 Screening of the mitochondrial gene in the patients with early-onset diabetes (cohort 1 and cohort 2)

Cohort 2 with 50 unrelated Chinese patients with EOD who had a maternally inherited diabetes (MID) family history were included from the inpatient and outpatient populations of the Department of Endocrinology and Metabolism at Peking University People’s Hospital from January 2013 to December 2017. Then, 40 unrelated EOD patients with MID were recruited to identify the potential disease-associated variants. Cohort 2 included the above 90 patients. All the patients were of northern Han Chinese ancestry and residents of Beijing. They have been diagnosed with diabetes according to the 1999 World Health Organization criteria (15). The inclusion criteria for the EOD patients were as follows: (1) diagnosed with T2DM ≤40 years, (2) patients with the typical clinical features of type 1 diabetes or other specific forms of diabetes (e.g., chronic pancreatitis) and those who were positive for anti-glutamic acid decarboxylase antibodies, anti-islet cell antibodies were excluded, and (3) without the m. A3243G variant. The clinical information of 90 EOD patients was collected, including demographics, initial presentation, the treatment of diabetes, physical examination results, and laboratory test results, which are summarized at the time of enrollment in **Table 1**. The patients were diagnosed as being overweight or obese, based on the criteria: overweight was defined as a body mass index (BMI) ≥24 kg/m^2^ and <28 kg/m^2^, and obesity was defined as a BMI ≥28 kg/m^2^. Coronary heart disease (CHD) was determined as a history of the disease. Cerebrovascular disease was determined as a history of transient ischemic attack or ischemic or hemorrhagic stroke. Hypertension was determined as a systolic blood pressure ≥140 mmHg and/or diastolic blood pressure ≥90 mmHg or the current treatment of hypertension. Dyslipidemia was defined as total cholesterol ≥5.18 mmol/L, triglycerides ≥1.70 mmol/L, LDL-c ≥3.37 mmol/L, HDL-c1.3 mmol/L in women or HDL-c<1.04 mmol/L in men, or treatment with antihyperlipidemic agents (16). Diabetic kidney disease (DKD) was defined as having a urinary albumin-to-creatinine ratio ≥30 mg/g or an estimated glomerular filtration rate (eGFR) <60 ml/min/1.73 m^2^. Patients diagnosed as having a urinary tract infection, other glomerular diseases, or gross hematuria were excluded. Homeostasis model assessment (HOMA) was used to evaluate β-cell function and insulin sensitivity. IR index (HOMA-IR) = [fasting insulin (Fins, mIU/L) × fasting plasma glucose (FPG, mmol/L)]/22.5, and β-cell function (HOMA-β) = [Fins (mIU/L) × 20]/[FPG (mmol/L) — 3.5] (17). When assessing the levels of HOMA-IR and HOMA-β, we excluded patients who were using insulin. MetS was assessed based on the International Diabetes Federation (IDF 2005) criteria: the diagnosis of central obesity (WC ≥ 90 cm in men or ≥ 80 cm in women), plus any two of the following four factors: (1) TG levels ≥ 1.7 mmol/L, (2) HDL-C < 1.03 mmol/L in men or < 1.29 mmol/L in women, (3) SBP ≥ 130 mmHg or DBP ≥ 85 mmHg or previous diagnosis with hypertension, (4) FBG ≥ 5.6 mmol/L, or previous diagnosis with type 2 diabetes mellitus (18).

**Table 1:** The clinical characteristics of the patients from cohort 1 and cohort 2.

Characteristics	cohort 1 (n=50)	cohort 2 (n=90)
Sex, male/female	35/15	59/31
Age, years	34.7 ± 8.4	35.3 ± 7.1
Age at diagnosis, years	29.9 ± 7.0	30.2 ± 6.0
Duration, years	3.5 (0.0, 7.0)	4.0 (1.0, 7.3)
BMI, kg/m^2^	28.5 ± 4.5	26.9 ± 4.2
WC, cm		
Male	99.1 ± 11.9	94.8 ± 11.1
Female	92.1 ± 11.8	88.8 ± 10.0
SBP, mmHg	130 ± 17	126 ± 16
DBP, mmHg	84 ± 11	83 ± 11
Laboratory examination		
FPG, mmol/l	9.1 ± 3.2	8.7 ± 3.5
FINS, μU/ml	16.2 (9.9, 27.8)	12.5 (7.8, 21.5)
HbA1c, %	8.9 ± 1.9	8.5 ± 2.1
LDL, mmol/l	2.7 ± 0.9	2.8 ± 0.8
HDL, mmol/l		
Male	0.9 (0.8, 1.0)	0.9 (0.8, 1.0)
Female	1.0 (0.9, 1.1)	1.0 (0.9, 1.2)
TC, mmol/l	4.9 ± 1.2	4.7 ± 1.1
TG, mmol/l	2.1 (1.4, 4.2)	1.8 (1.2, 2.8)
UA, umol/l		
Male	393.2 ± 80.3	374.5 ± 82.2
Female	337.8 ± 75.2	328.0 ± 86.9
ALT, U/l	29.5 (19.5, 46.8)	24.0 (16.0, 40.0)
AST, U/l	22.5 (18.0, 32.5)	20.0 (16.0, 27.5)
CRE, µmol/l	66.8 ± 28.1	63.7 ± 23.3
eGFR, ml/min/1.73 m^2^	167.9 ± 54.9	158.7 ± 46.8
ACR, mg/g	74.5 (9.7, 190.8)	17.8 (3.0, 93.8)
DKD (n, %)	35 (70.0)	40 (44.4)
DR (n, %)	12 (24.0)	17 (18.9)
Family history of MID (n, %)	50 (100.0)	90 (100.0)

BMI, body mass index; WC, waist circumference; SBP, systolic blood pressure; DBP, diastolic blood pressure; FPG, fasting plasmatic glucose; FINS, fasting insulin; HbA1c, hemoglobin A1c; LDL, low-density lipoprotein; HDL, high-density lipoprotein; TC, total cholesterol; TG, triacylglycerol; UA, uric acid; CRE, creatinine; eGFR, estimated glomerular filtration rate; ACR, albumin-to-creatinine ratio; DKD, diabetic kidney disease; DR, diabetic retinopathy; MID, maternal inherited diabetes.

Values are means ± SDs, and medians (interquartile range) were for non-normally distributed data.

### 2.3 The methods for screening the mitochondrial gene in cohort 1 and cohort 2

DNA was extracted from peripheral blood using standard procedures. The whole mitochondrial DNA of 50 patients in cohort 1 was used with NGS. For them, the Agilent Sureselect XT2 capture chip was used for the mitochondrial gene (accession number: NC_012920.1). A HiSeq 2500 Sequencing System (Illumina, San Diego, CA, USA) was adopted for sequencing. Sequencing data were obtained with the target region coverage (~100%) and the sequencing depth on target > 2,000. All rare variants of mtDNA were validated by Sanger sequencing.

### 2.4 Annotation and filtration of the mitochondrial DNA variants possibly associated with mitochondrial diseases

The criteria for selecting the mtDNA variants possibly associated with mitochondrial diseases from the NGS data were as follows: (1) variant frequency in the cohort 1 was <20%; (2) sequencing depth >400 ×; (3) we searched the variants in the MITOMAP database (https://www.mitomap.org/MITOMAP) and selected the mtDNA variants that were possibly disease related based on the database. (4) The potential disease-related variants were validated by Sanger sequencing.

### 2.5 Screening of potential disease-related variants from cohort 1 in cohort 2

The potential disease-related variants screened and identified from cohort 1 were verified in cohort 2 by Sanger sequencing. PCR amplification was performed using PCR Enzyme Mix. The 24 pairs of mitochondrial primers are shown in **Supplementary Table 1**. The conditions for the PCR were as follows: initial denaturation at 94°C for 1 min, followed by 35 cycles of denaturation at 94°C for 30 s, primer annealing for 45 s at 61°C, and primer extension at 72°C for 2 min. A final extension was at 72°C for 5 min. The direct sequencing of PCR products was purified using a gel extraction kit (Axygen, California, USA) and performed with the ABI 3730xl DNA sequencer (Applied Biosystems) (19).

### 2.6 The analysis of sequencing data from cohort 2

The sequencing data from cohort 2 were analyzed. The pathogenicity of these variants in the coding area and tRNA were evaluated by HmtVar (https://www.hmtvar.uniba.it/). The pathogenicity of non-synonymous CDS variants were classified into four categories: (1) polymorphic: disease score (DS) < 0.43, allele frequency (AF) > 0.003264; (2) likely polymorphic: DS < 0.43, AF ≤ 0.003264; (3) likely pathogenic: DS ≥ 0.43, AF > 0.003264; and (4) pathogenic: DS ≥ 0.43, AF ≤ 0.003264. The pathogenicity of tRNA variants was classified into four categories: (1) polymorphic: DS < 0.35, AF > 0.005020; (2) likely polymorphic: DS < 0.35, AF ≤ 0.005020; (3) likely pathogenic: DS ≥ 0.35, AF > 0.005020; (4) pathogenic: DS ≥ 0.35, AF ≤ 0.005020. The DS was evaluated according to the software of MutPred, Polyphen2, Panther, PhD SNP, and SNPs & GO.

### 2.7 Screening for the potential disease-related variants in the expanded samples (cohort 3)

#### 2.7.1 The participants for the case–control study (cohort 3)

A total of 1,571 T2DM patients and 496 healthy individuals with NGT were also enrolled in the case–control study. The inclusion criteria for the control samples were as follows: normal glucose tolerance confirmed *via* a 75 g oral glucose tolerance test according to the 1999 World Health Organization criteria (15); at the age of over 40; and hemoglobin A1c (HbA1c) <6%. Their clinical features are also summarized in **Table 2**.

**Table 2:** Characteristics of the subjects of type 2 diabetes or normal glucose tolerance in cohort 3.

Characteristic	Controlsn=496	Patients with DM n=1,571	P-value
Sex, male/female	248/248	898/673	0.005
Age, years	52.0 ± 8.6	52.6 ± 11.8	0.247
Duration, years	–	1.0 (0.1, 6.0)	–
BMI, kg/m^2^	25.7 ± 3.3	25.8 ± 3.5	0.808
WC, cm			
Male	89.5 ± 8.9	92.8 ± 10.3	<0.001
Female	84.8 ± 9.3	87.8 ± 9.7	<0.001
SBP, mmHg	131 ± 17	129 ± 16	0.003
DBP, mmHg	86 ± 12	83 ± 13	<0.001
Hypertension	136 (27.5)	319 (50.9)	0.000
Dyslipidemia	387 (78.0)	734 (87.3)	0.000
Laboratory examination			
FPG, mmol/l	5.4 ± 0.4	8.7 ± 2.4	<0.001
FINS, μU/ml	7.1 (4.9, 10.2)	8.8 (5.9, 13.6)	<0.001
HbA1c, %	5.4 ± 0.3	8.2 ± 1.9	<0.001
LDL, mmol/l	2.9 ± 0.7	3.0 ± 0.9	0.085
HDL, mmol/l			
Male	1.1 ± 0.3	1.0 ± 0.3	0.005
Female	1.2 ± 0.2	1.2 ± 0.3	0.848
TC, mmol/l	4.9 ± 0.8	4.9 ± 1.3	0.467
TG, mmol/l	1.1 (0.7, 1.6)	1.7 (1.3, 2.5)	<0.001
UA, μmol/l	269 ± 74	327 ± 91	0.000
Male	307.0 ± 71.3	347.5 ± 89.9	<0.001
Female	231.7 ± 54.9	296.6 ± 82.9	<0.001
ALT, U/l	19.0 (14.3, 25.0)	22.0 (16.0, 35.0)	<0.001
AST, U/l	21.0 (18.0, 24.0)	1.7 (1.3, 2.5)	0931
CRE, µmol/l	61.9 ± 28.3	65.7 ± 18.8	0.003
eGFR, ml/min/1.73 m^2^	129.3 ± 31.4	125.5 ± 37.5	0.063
HOMA-IR	1.7 (1.1, 2.4)	3.3 (2.1, 5.1)	<0.001

BMI, body mass index; WC, waist circumference; SBP, systolic blood pressure; DBP, diastolic blood pressure; FPG, fasting plasmatic glucose; FINS, fasting insulin; HbA1c, hemoglobin A1c; LDL, low-density lipoprotein; HDL, high-density lipoprotein; TC, total cholesterol; TG, triacylglycerol; UA, urine acid; CRE, creatinine; eGFR, estimated glomerular filtration rate; HOMA-IR, homeostasis model assessment insulin resistance index.

Values are means ± SDs or numbers of subjects (percentages), and medians (interquartile range) were for non-normally distributed data. Student’s t-tests, and chi-square tests were performed to compare the clinical difference between two groups. The Mann–Whitney rank-sum test was used to make comparisons for the non-normally distributed variable.

#### 2.7.2 The methods for genotyping the mtDNA in cohort 3

The above selected mtDNA variants were screened in the case–control cohort (cohort 3) using high-throughput genotyping on a MassARRAY platform (Sequenom) to genotype the mtDNA variants. Duplicate DNA samples and wild genotypes confirmed by sequencing were simultaneously genotyped as quality controls.

### 2.8 Statistical analysis

Statistical tests were performed with the SPSS software 23.0 (Chicago, IL, USA). Continuous variables are presented as the means and standard deviations (means ± standard deviations) for normally distributed data or as medians (interquartile range) for non-normally distributed data. Categorical variables are presented as numbers and percentages. Student’s *t*-tests, Fisher’s exact tests, and chi-square tests were performed to compare the clinical difference. The Mann–Whitney rank-sum test was used to make comparisons for the non-normally distributed variable. When we analyzed the metabolic features between patients with 42 variants and without those variants in cohort 2 and cohort 3, the levels of FINS, HOMA-IR, and HOMA-β were grouped according to the tertiles. An ordered-classification chi-square test was used to compare the differences between groups. Logistic regression analysis was conducted to address the relation between variables. *P* < 0.05 was considered statistically significant.

## 3 Results

### 3.1 Characteristics of the study participants

The clinical characteristics of the enrolled participants are shown in **Table 1**. The whole mitochondrial genome was screened by NGS in cohort 1. In addition, the cohort 2 including 90 EOD patients was sequenced to analyze the variants possibly associated with metabolic diseases. The 90 patients had an MID family history, of which 59 (65.6%) were men and the average age at diagnosis was 30.2 ± 6.0 years. The mean BMI was 26.9 ± 4.2 kg/m^2^, and the average HbA1c was 8.5 ± 2.1%. The clinical characteristic features of cohort 3 are shown in **Table 2**.

### 3.2 The analysis of mtDNA variants in the mitochondrial gene in cohort 1 and cohort 2

Through the whole mitochondrial genome sequencing of 50 patients with MID, the mean coverage of the target genes was above 95.0%, and the mean depth was above 400 ×. In total, 474 variants on mtDNA were identified, including 225 variants in the coding region and 249 variants in the non-coding region. Among the coding region, 150 synonymous variants, one non-sense variant, and 74 non-synonymous variants were identified. According to the filtering strategy in the method, 42 mtDNA variants are identified in **Table 3**. Then, these 42 mtDNA variants were screened in cohort 2, and the frequency of these variants in cohort 2 are displayed in **Table 3**. Among them, seven variants were located in the D-loop, four variants in MT-ND1, two variants in MT-ND2, one in MT-CO2, two in MT-ATP6, two variants in MT-ND3, three variants in MT-ND4, five variants in MT-ND5, and one variant in MT-ND6. In addition, three variants were in MT-CYB, and six variants in tRNA, and six variants were in rRNA. The above 42 variants were located in complex I, complex III, complex IV, complex V and a non-coding area (including ND6 and D-loop), tRNA, and rRNA.

**Table 3:** The rare mitochondrial DNA (mtDNA) variants from cohort 2 possibly associated with mitochondrial disease (n=42).

No.	Base change	Frequency in cohort 2	Amino acid change	hom/het	Locus Type	Locus	Prediction	Associated disease	Reference
1	C150T	8 (8.9)	/	hom	MT-DLOOP	/	/	Longevity/cervical carcinoma/HPV infection risk	(20, 21)
2	T195C	4 (4.3)	/	hom	MT-DLOOP	/	/	BD-associated/melanoma	(22, 23)
3	A663G	4 (4.3)	/	hom	rRNA	MT-RNR1	/	Coronary atherosclerosis risk	(24)
4	A827G	2 (2.2)	/	hom	rRNA	MT-RNR1	/	Deaf	(25)
5	T1005C	4 (4.4)	/	hom	rRNA	MT-RNR1	/	Deaf	(26)
6	C1048T	2 (2.2)	/	hom	rRNA	MT-RNR1	/	Deaf	(27)
7	G1719A	1 (1.1)	/	hom	rRNA	MT-RNR2	/	Ischemic stroke	(28)
8	C2835T	1 (1.1)	/	hom	rRNA	MT-RNR2	/	Rett syndrome	(29, 30)
9	A3397G	2 (2.2)	Met31Val	hom	CDS	MT-ND1	Likely Polymorphic	AD, PD	(31–33)
10	T3398C	2 (2.2)	Met31Thr	hom	CDS	MT-ND1	Polymorphic	DM/GDM/possibly LVNC cardiomyopathy-associated	(34–36)
11	C3497T	2 (2.2)	Ala64Val	hom	CDS	MT-ND1	Likely Polymorphic	LHON	(33)
12	A3943G	1 (1.1)	Iie213Val	hom	CDS	MT-ND1	Likely Polymorphic	Aging	(37)
13	A4833G	4 (4.4)	Thr122Ala	hom	CDS	MT-ND2	Likely Pathogenic	DM; AD, PD	(38, 39)
14	A5466G	1 (1.1)	Thr333Ala	hom	CDS	MT-ND2	Likely Polymorphic	DM; deafness; cardiomyopathy	(40)
15	G7598A	1(1.1)	Ala5Thr	hom	CDS	MT-CO2	Polymorphic	Possible LHON helper variant; Deafness	(41, 42)
16	C8414T	9 (1.0)	Leu17Phe	hom	CDS	MT-ATP6	Likely Pathogenic	Longevity	(43)
17	C8794T	3 (3.3)	His90Tyr	hom	CDS	MT-ATP6	Polymorphic	Exercise endurance/coronary Atherosclerosis risk	(24, 44)
18	A10005G	1 (1.1)	/	hom	tRNA	MT-TG	Likely Polymorphic	Hearing loss	(45)
19	T10084C	1 (1.1)	Iie9Thr	hom	CDS	MT-ND3	Polymorphic	Mitochondrial encephalomyopathy	(46)
20	A10086G	1 (1.1)	Asn10Asp	hom	CDS	MT-ND3	Likely Pathogenic	Hypertensive end-stage renal disease	(47)
21	A11084G	1 (1.1)	Thr109Ala	hom	CDS	MT-ND4	Pathogenic	MELAS	(46)
22	T11204C	1 (1.1)	Phe149Leu	hom	CDS	MT-ND4	Likely Polymorphic	Head and neck cancer	(48)
23	A12026G	1 (1.1)	Ile423Val	hom	CDS	MT-ND4	Polymorphic	DM	(49)
24	G12192A	1 (1.1)	/	hom	tRNA	MT-TH	Likely Polymorphic	Cardiomyopathy; LHON; deaf	(50, 51)
25	T12338C	3 (3.3)	Met1Thr	hom	CDS	MT-ND5	Likely Pathogenic	Lebers optic atrophycardiomyopathy	(52–54)
26	A12361G	2 (2.2)	Thr9Ala	hom	CDS	MT-ND5	Polymorphic	Non-alcoholic fatty liver disease	(4)
27	T12811C	1 (1.1)	Tyr159His	hom	CDS	MT-ND5	Polymorphic	LHON	(55)
28	G13135A	1 (1.1)	Ala267Thr	hom	CDS	MT-ND5	Polymorphic	HCM	(56)
29	G13708A	3 (3.3)	Ala458Thr	hom	CDS	MT-ND5	Polymorphic	LHON	(57)
30	T14502C	1 (1.1)	Ile58Val	hom	CDS	MT-ND6	Likely Polymorphic	LHON	(58)
31	T14674C	1 (1.1)	/	hom	tRNA	MT-TE	Pathogenic	Reversible COX deficiency myopathy	(59)
32	A14693G	3 (3.3)	/	hom	tRNA	MT-TE	Likely Polymorphic	MELAS; LHON; deaf	(60–62)
33	A14927G	1 (1.1)	Thr61Ala	hom	CDS	MT-CYB	Likely Polymorphic	Cardiomyopathy	(63)
34	A15218G	1 (1.1)	Thr158Ala	hom	CDS	MT-CYB	Likely Pathogenic	Possible LHON modulator; epilepsy, sensorineural hearing impairment or DM	(64, 65)
35	G15497A	1 (1.1)	Gly251Ser	hom	CDS	MT-CYB	Polymorphic	Obesity	(66)
36	G15927A	2 (2.2)	/	hom	tRNA	MT-TT	Likely Pathogenic	Deafness	(54)
37	A15951G	1 (1.1)	/	hom	tRNA	MT-TT	Polymorphic	LHON	(47, 67)
38	T16093C	4 (4.4)	/	hom	Regulatory Sequence	MT-DLOOP	/	Breast cancer	(68)
39	G16129A	7 (7.8)	/	hom	Regulatory Sequence	MT-DLOOP	/	Breast cancer	(68)
40	A16183C	2 (2.2)	/	het	Regulatory Sequence	MT-DLOOP	/	Breast cancer; hypertension	(68, 69)
41	C16192T	1 (1.1)	/	hom	Regulatory Sequence	MT-DLOOP	/	Malignant melanoma	(22)
42	A16300G	1 (1.1)	/	hom	Regulatory Sequence	MT-DLOOP	/	Head/neck tumor	(70)

Het, heterozygote; Hom, homozygote; BD, bipolar disorder; AD, Alzheimer disease; PD, Parkinson disease; DM, diabetes mellitus; GDM, gestational diabetes mellitus; LVNC, left ventricular non-compaction; LHON, Leber hereditary optic neuropathy; MELAS, mitochondrial encephalopathy lactic acidosis and stroke-like episodes; HCM, hypertrophic cardiomyopathy.

**Figure 2:**
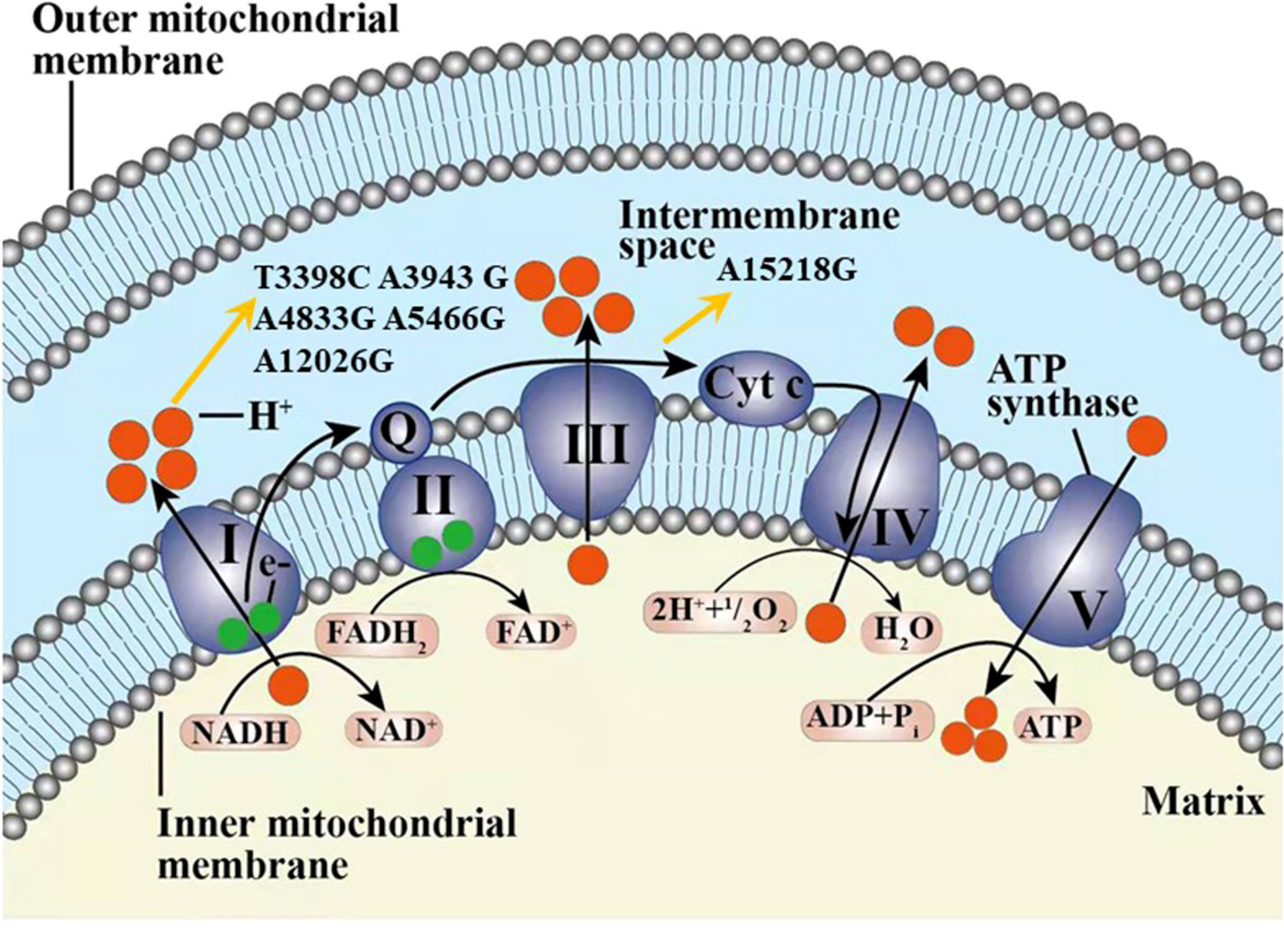
The six variants from cohort 2 and one variant from cohort 3 found in our study possibly associated with diabetes in our study displayed in the electron transport chain of mitochondria, including T3398C, A3943G, A4833G, A5466G, A12026G, and A15218G. The complexes I-V marked as I-V were included in the respiratory chain and oxidative phosphorylation system. Q, coenzyme Q10; Cyt c, cytochrome c; e-, electron.

Among the 42 mtDNA variants, five variants (m.T3398C, m.A4833G, m.A5466G, m.A12026G, m.A15218G) have been reported to be possibly associated with diabetes (**Figure 2**).

### 3.3 The comparison of metabolic features between patients with 42 variants and without those variants in cohort 2

Compared with the patients without 42 variants, the patients with these variants had higher levels of FINS and HOMA-IR with a significant difference [14.6 (10.0, 26.7) vs. 8.1 (5.8, 13.4), *P* = 0.006; 6.5 (3.2, 10.2) vs. 3.3 (1.8, 4.3), *P* = 0.002]. There was no difference between the two groups of BMI, waist circumference, and HbA1c. In addition, the prevalence of DKD was significantly higher in the group with the variants than those without the variants (48.4% vs. 20.0%, P = 0.001) in **Table 4**.

**Table 4:** Comparison of subjects with the 42 variants of mtDNA with the subjects without those variants in cohort 2.

Characteristics	without variants n=25	with variants n=65	P
Sex, male/female	13/12	47/28	0.067
Age, years	35.2 ± 6.4	35.4 ± 7.4	0.901
BMI, kg/m^2^	25.5 ± 2.7	27.4 ± 4.5	0.060
WC, cm			
Male	93.0 ± 10.8	95.4 ± 11.2	0.505
Female	86.5 ± 5.5	90.1 ± 11.9	0.351
SBP, mmHg	115 ± 17	112 ± 26	0.690
DBP, mmHg	86 ± 11	86 ± 13	0.106
Hypertension, n (%)	13 (23.6)	15 (23.1)	0.942
MetS, n (%)	27 (49.1)	38 (58.5)	0.305
DR, n (%)	4 (16.7)	13 (21.0)	0.653
DKD, n (%)	11 (20.0)	31 (48.4)	0.001
FPG, mmol/l	8.2 ± 3.5	8.8 ± 3.5	0.464
FINS, μU/ml	8.1 (5.8, 13.4)	14.6 (10.0, 26.7)	0.006
HbA1c, %	8.4 ± 2.0	8.5 ± 2.1	0.872
LDL, mmol/l	3.0 ± 0.8	2.7 ± 0.8	0.133
HDL, mmol/l			
Male	0.9 ± 0.2	1.0 ± 0.3	0.486
Female	1.1 ± 0.2	1.0 ± 0.2	0.256
TC, mmol/l	5.0 ± 1.1	4.8 ± 0.9	0.368
TG, mmol/l	1.9 (1.0, 2.6)	1.8 (1.2, 3.3)	0.692
UA, μmol/l			
Male	377.8 ± 85.2	373.6 ± 82.3	0.873
Female	323.0 ± 105.0	331.4 ± 75.7	0.801
ALT, U/l	19.5 (12.5, 34.5)	25.0 (17.0, 29.5)	0.253
AST, U/l	18.0 (15.3, 24.8)	21.0 (17.0, 29.5)	0.093
CRE, µmol/l	57.1 ± 16.0	66.2 ± 25.2	0.096
eGFR, ml/min*1.73m^2^	156.5 ± 37.5	159.5 ± 50.2	0.788
ACR, mg/g	7.9 (1.9, 65.2)	21.8 (3.0, 106.4)	0.077
HOMA-IR	3.3 (1.8, 4.3)	6.5 (3.2, 10.2)	0.002
HOMA-β	61.1 (24.0, 110.7)	72.8 (38.3, 120.6)	0.126

BMI, body mass index; WC, waist circumference; SBP, systolic blood pressure; DBP, diastolic blood pressure; MetS, metabolic syndrome; FPG, fasting plasmatic glucose; FINS, fasting insulin; HbA1c, hemoglobin A1c; LDL, low-density lipoprotein; HDL, high-density lipoprotein; TC, total cholesterol; TG, triacylglycerol; UA, uric acid; CRE, creatinine; eGFR, estimated glomerular filtration rate; ACR, albumin-to-creatinine ratio; DKD, diabetic kidney disease; DR, diabetic retinopathy. HOMA-IR, homeostasis model assessment insulin resistance index. HOMA-β, homeostasis model assessment β-cell function.

Values are means ± SDs or numbers of subjects (percentages), and medians (interquartile range) were for non-normally distributed data. Student’s t-tests, and chi-square tests were performed to compare the clinical difference between two groups. The Mann–Whitney rank-sum test was used to make comparisons for the non-normally distributed variable.

A total of 42 mtDNA variants were in different domains of mitochondria including complex I, complex III, complex IV, complex V, non-coding region, rRNA, and tRNA. Patients with variants in the non-coding region had a higher percentage of obesity and levels of FINS than those without these variants with significant difference (62.1% vs. 24.6%, P = 0.001; 80.0% vs. 26.5% P < 0.001). The patients with the variants in rRNA had a higher prevalence of obesity (71.4% vs. 30.3%, P = 0.007), and the patients with the variants in complex I had a higher level of FINS (64.3% vs. 34.3%, P = 0.049). There was no significant difference on the frequency of different BMIs and levels of FINS, HOMA-IR, and HOMA-β of the patients with variants in other domains of mitochondria in **Tables 5**, **6**.

**Table 5:** Comparison of body mass index (BMI) and other traits among the patients with variants and without these variants in the complex I, III, IV, and V domains of mitochondria.

	Tertile/Category	In Complex I		P-value	In Complex III		P- value	In Complex IV		P-value	In Complex V		P-value
		Without Variants	With variants		Without Variants	With variants		Without Variants	With variants		Without Variants	With variants	
BMI	normal	14/59 (23.7%)	7/31 (22.6%)	0.964	20/85 (23.5%)	1/5 (20.0%)	0.841	21/88 (23.9%)	0/2 (0.0%)	1.000	18/72 (25.0%)	3/18 (16.7%)	0.057
	overweight	23/59 (39.0%)	13/31 (41.9%)		33/85 (38.8%)	3/5 (60.0%)		35/88 (39.8%)	1/2 (50.0%)		32/72 (44.4%)	4/18 (22.2%)	
	obesity	22/59 (37.3%)	11/31 (35.5%)		32/85 (37.6%)	1/5 (20.0%)		32/88 (36.4%)	1/2 (50.0%)		22/72 (30.6%)	11/18 (61.1%)	
FINS*	T1	9/35 (25.7%)	0/14 (0.0%)	0.049	0/46 (0.0%)	0/3 (0.0%)	0.208	9/47 (19.1%)	0/2 (0.0%)	1.000	8/38 (21.1%)	1/11 (9.1%)	0.677
	T2	14/35 (40.0%)	5/14 (35.7%)		19/46 (41.3%)	0/3 (0.0%)		18/47 (38.3%)	1/2 (50.0%)		15/38 (39.5%)	4/11 (36.4%)	
	T3	12/35 (34.3%)	9/14 (64.3%)		18/46 (39.1%)	3/3 (100.0%)		20/47 (42.6%)	1/2 (50.0%)		15/38 (39.5%)	6/11 (54.5%)	
HOMA-IR*	T1	7/35 (20.0%)	0/13 (0.0%)	0.131	7/45 (15.6%)	0/3 (0.0%)	0.375	7/46 (15.2%)	0/2 (0.0%)	1.000	6/37 (16.2%)	1/11 (9.1%)	0.264
	T2	13/35 (37.1%)	4/13 (30.8%)		17/45 (37.8%)	0/3 (0.0%)		16/46 (34.8%)	1/2 (50.0%)		15/37 (40.5%)	2/11 (18.2%)	
	T3	15/35 (42.9%)	9/13 (69.2%)		21/45 (46.7%)	3/3 (100.0%)		23/46 (50.0%)	1/2 (50.0%)		16/37 (43.2%)	8/11 (72.7%)	
HOMA-β*	T1	17/35 (48.6%)	2/13 (15.4%)	0.101	18/45 (40.0%)	1/3 (33.3%)	1.000	19/46 (41.3%)	0/2 (0.0%)	0.512	13/37 (35.1%)	6/11 (54.5%)	0.125
	T2	8/35 (22.9%)	5/13 (38.5%)		12/45 (26.7%)	1/3 (33.3%)		12/46 (26.1%)	1/2 (50.0%)		9/37 (24.3%)	4/11 (36.4%)	
	T3	10/35 (28.6%)	6/13 (46.2%)		15/45 (33.3%)	1/3 (33.3%)		15/46 (32.6%)	1/2 (50.0%)		15/37 (40.5%)	1/11 (9.1%)	

BMI, body mass index; FINS, fasting insulin; HOMA-IR, homeostasis model assessment insulin resistance index; HOMA-β, homeostasis model assessment β-cell function; T, tertile.

T1–3 were grouped according to tertile category of FINS, HOMA-IR. Ordered classification chi-square test was used to compare the differences between groups. When we analyzed the difference of FINS and HOMA-IR, patients using insulin were excluded. * means that the subjects who were treated with insulin were excluded. A P-value <0.05 was considered significant.

**Table 6:** Comparison of BMI and other traits among the patients with variants and without these variants in the non-coding region, transfer RNA, and rRNA of mitochondria.

	Tertile/Category	In non-coding		P-value	In rRNA		P-value	In tRNA		P-value
		Without variants	With Variants		Without variants	With Variant		Without variants	With Variants	
BMI	normal	15/61 (24.6%)	6/29 (20.7%)	0.001	18/76 (23.7%)	3/14 (21.4%)	0.007	19/79 (24.1%)	2/11 (18.2%)	0.799
	overweight	31/61 (50.8%)	5/29 (17.2%)		35/76 (46.1%)	1/14 (7.1%)		32/79 (40.5%)	4/11 (36.4%)	
	obesity	15/61 (24.6%)	18/29 (62.1%)		23/76 (30.3%)	10/14 (71.4%)		28/79 (35.4%)	5/11 (45.5%)	
FINS*	T1	6/34 (17.6%)	3/15 (20.0%)	<0.001	8/39 (20.5%)	1/10 (10.0%)	0.522	8/42 (19.0%)	1/7 (14.3%)	0.317
	T2	19/34 (55.9%)	0/15 (0.0%)		16/39 (41.0%)	3/10 (30.0%)		18/42 (42.9%)	1/7 (14.3%)	
	T3	9/34 (26.5%)	12/15 (80.0%)		15/39 (38.5%)	6/10 (60.0%)		16/42 (38.1%)	5/7 (71.4%)	
HOMA-IR*	T1	5/33 (15.2%)	2/15 (13.3%)	0.064	7/38 (18.4%)	0/10 (0.0%)	0.272	6/41 (14.6%)	1/7 (14.3%)	0.056
	T2	15/33 (45.5%)	2/15 13.3%		14/38 (36.8%)	3/10 (30.0%)		17/41 (41.5%)	0/7 (0.0%)	
	T3	13/33 (39.4%)	11/15 73.3%		17/38 (44.7%)	7/10 (70.0%)		18/41 (43.9%)	6/7 (85.7%)	
HOMA-β*	T1	17/38 (44.7%)	2/10 (20.0%)	0.364	17/38 (44.7%)	2/10 (20.0%)	0.364	16/41 (39.0%)	3/7 (42.9%)	1.000
	T2	9/38 (23.7%)	4/10 (40.0%)		9/38 (23.7%)	4/10 (40.0%)		11/41 (26.8%)	2/7 (28.6%)	
	T3	12/38 (31.6%)	4/10 (40.0%)		12/38 (31.6%)	4/10 (40.0%)		14/41 (34.1%)	2/7 (28.6%)	

BMI, body mass index; FINS, fasting insulin; HOMA-IR, homeostasis model assessment insulin resistance index; HOMA-β, homeostasis model assessment β-cell function; T, tertile.

T1–3 were grouped according to the tertile category of FINS and HOMA-IR. Ordered-classification chi-square test was used to compare the differences between groups. When we analyzed the difference of FINS and HOMA-IR, patients using insulin were excluded. * means that the subjects who were treated with insulin were excluded. A P-value <0.05 was considered significant.

### 3.4 The high-throughput genotyping results of mtDNA variants in cohort 3

**Figure 3:**
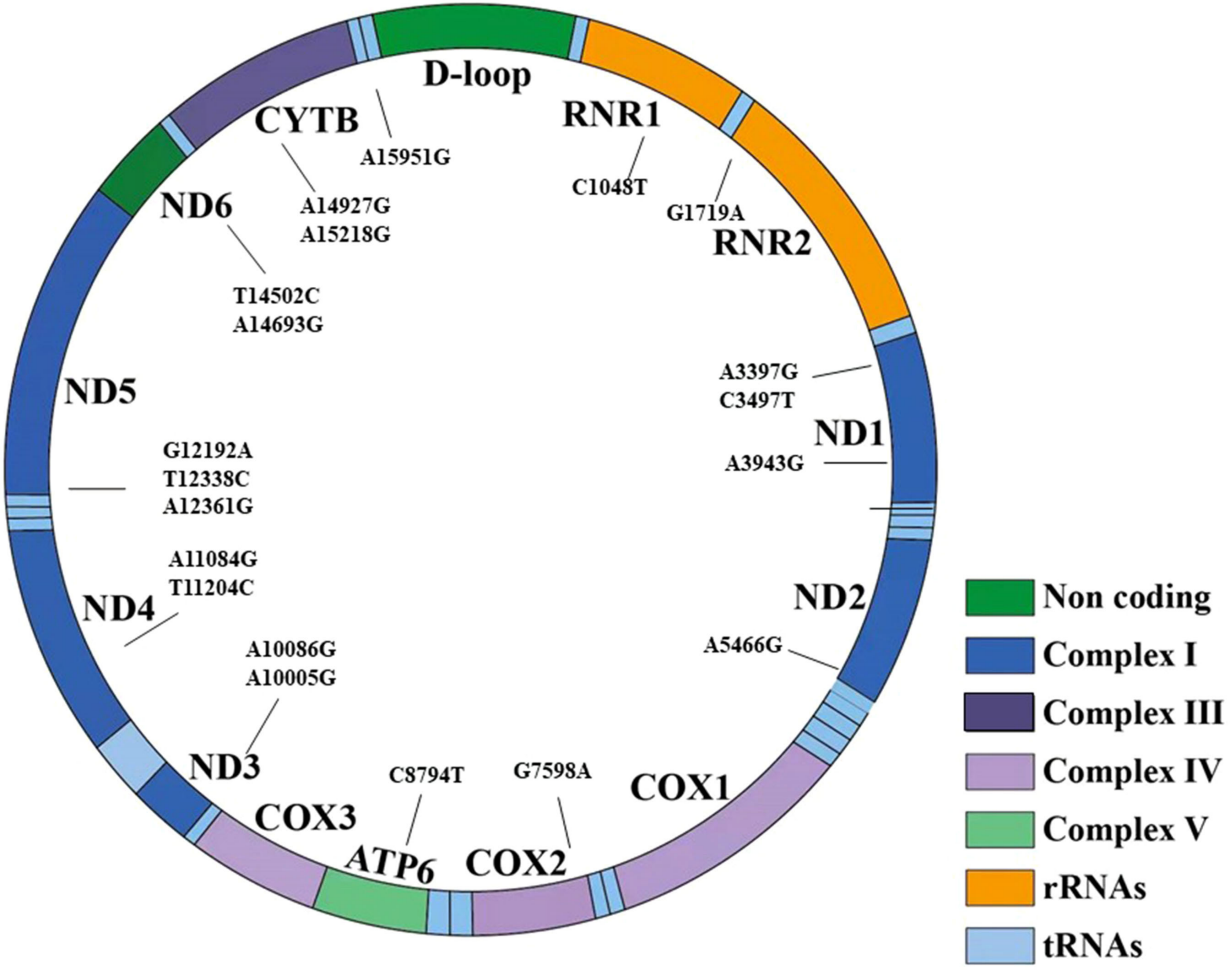
The 20 variants have been shown in the structure of the mitochondrial genes and different domains. It included seven regions: non-coding region, complex I, complex III, complex IV, complex V, transfer RNA, and rRNA.

In cohort 3, 42 mtDNA variants were screened using high-throughput genotyping, to investigate the phenotype and genotype of mtDNA variants. After the sequencing, 20 homogeneous variants associated with mitochondrial diseases were successfully captured and met the criteria of quality control in **Table 7** and **Figure 3**. Excluding the variant of m.C3497T, there was no significant difference on the frequency of the mtDNA variants in the diabetic group and NGT group. We further enlarged 200 NGT subjects to screen the m.C3497T variant and found that there was no significant difference between the two groups.

**Table 7:** The analysis of 20 mtDNA variants in the case–control study from cohort 3.

No.	Variant	Position	Neurodisease	Amino change	Locus type	Locus	Prediction	Genotype distribution		MAF %		P-value *	Adjusted OR (95% CI) *
								NGT group	No. of diabetic patients of variant genotype/wild genotype	No. of NGT group of variant genotype/wild genotype	DM group		
1	C1048T	1048	No	–	rRNA	MT-RNR1	Unavailable	31/1,499	3/449	2	0.7	0.063	3.095 (0.942,10.172)
2	G1719A	1719	No	–	rRNA	MT-RNR1	Unavailable	22/1,517	10/474	1.4	2.1	0.330	0.687 (0.323, 1.462)
3	A3397G	3397	Yes	Met31Val	CDS	MT-ND1	Likely Polymorphic	14/1,492	1/494	0.9	0.2	0.139	4.635 (0.608, 35.340)
4	C3497T	3497	Yes	Ala64Val	CDS	MT-ND1	Likely Polymorphic	34/1,530	19/474	2.2	3.9	0.043	0.554 (0.313-0.981)
5	A3943G	3943	No	Iie213Val	CDS	MT-ND1	Likely Polymorphic	1/1,567	0/495	0.06	0	1.000	–
6	A5466G	5466	No	Thr333Ala	CDS	MT-ND2	Likely Polymorphic	12/1,555	1/495	0.8	0.2	0.198	3.820 (0.495, 29.451)
7	G7598A	7598	Yes	Ala-5Thr	CDS	MT-CO2	Polymorphic	8/1,539	1/493	0.5	0.2	0.376	2.563 (0.320, 20.540)
8	C8794T	8794	No	His-90Tyr	CDS	MT-ATP6	Polymorphic	117/1,445	41/452	7.5	8.3	0.549	0.893 (0.616, 1.294)
9	A10005G	10005	No	–	tRNA	MT-TG	Likely Polymorphic	1/1,514	0/493	0.1	0	1.000	–
10	A10086G	10086	No	Asn10Asp	CDS	MT-ND3	Likely Pathogenic	1/1,537	2/492	0.1	0.4	0.135	0.160 (0.014, 1.769)
11	A11084G	11084	Yes	Thr109Ala	CDS	MT-ND4	Pathogenic	12/1,499	9/483	0.8	1.8	0.073	0.449 (0.187,1.078)
12	T11204C	11204	No	Phe149Leu	CDS	MT-ND4	Likely Polymorphic	5/1,540	4/491	0.3	0.8	0.172	0.399 (0.107, 1.490)
13	G12192A	12192	No	–	tRNA	MT-TH	Likely Polymorphic	4/1,553	4/492	0.3	0.8	0.105	0.317 (0.079, 1,271)
14	T12338C	12338	Yes	Met1Thr	CDS	MT-ND5	Likely Pathogenic	70/1,400	21/472	4.8	4.3	0.646	1.124 (0.683, 1.850)
15	A12361G	12361	No	Thr9Ala	CDS	MT-ND5	Polymorphic	58/1,507	13/481	3.7	2.6	0.256	1.424 (0.774, 2.621)
16	T14502C	14502	Yes	Iie58Val	CDS	MT-ND6	Likely Polymorphic	30/1,506	6/489	2	1.2	0.282	1.624 (0.672, 3.924)
17	A14693G	14693	Yes	–	tRNA	MT-TE	Likely Polymorphic	18/1,545	6/490	1.2	1.2	0.916	0.951 (0.376, 2.410)
18	A14927G	14927	No	Thr61Ala	CDS	MT-CYB	Likely Polymorphic	20/1,545	7/489	1.3	1.4	0.820	0.904 (0.380, 2.151)
19	A15218G	15218	Yes	Thr158Ala	CDS	MT-CYB	Likely Pathogenic	26/1,513	7/486	1.7	1.4	0.681	1.193 (0.515, 2.766)
20	A15951G	15951	Yes	–	tRNA	MT-TT	Polymorphic	16/1,526	6/489	1	1.2	0.744	1.170 (0.455, 3.007)

NGT, normal glucose tolerance; MAF, major allele frequency.

Logistic regression analysis was used to compare the prevalence of mtDNA variants in diabetic patients and the subjects with normal glucose tolerance. *means that the results were analyzed by logistic regression with no adjustment.

Among the 20 homogeneous variants, two variants of m.A3943G (**Figure 3**) and m.A10005G were just found in the diabetic group and did not exist in the NGT group. The detailed clinical features are shown in **Supplementary Table 2**.

### 3.5 The comparison of metabolic features between patients with the 20 mtDNA variants and without those variants in diabetic patients from cohort 3

In the diabetic group, the levels of FINS, BMI, HOMA-IR, and HOMA-β were similar between the group with the 20 variants and those without the variants. The prevalence of MetS was similar in the two groups (56.8% vs. 57.2%, *P* = 0.886). The prevalence of DKD was significantly higher in the group with the variants than those without the variants (18.7% vs. 14.6%, *P* = 0.049) in **Table 8**.

**Table 8:** The comparison of the subjects without the 20 variants and with the 20 variants in normal glucose tolerance and diabetes mellitus groups from cohort 3.

Characteristics	NGT (n=496)			DM (n=1,571)		
	without variantsn=364	with variantsn=132	p	without variantsn=1,159	with variantsn=412	p
Sex, male/female	173/191	75/57	0.067	657/502	241/171	0.524
Age, years	52.1 ± 8.9	51.5 ± 8.0	0.468	52.6 ± 11.8	52.6 ± 11.8	0.980
BMI, kg/m^2^	25.6 ± 3.3	26.1 ± 3.1	0.139	25.8 ± 3.5	25.7 ± 3.5	0.647
WC, cm						
Male	88.4 ± 8.3	91.2 ± 9.0	0.015	91.9 ± 10.0	92.0 ± 10.5	0.955
Female	84.6 ± 9.0	86.7 ± 8.4	0.12	89.4 ± 7.7	89.6 ± 8.7	0.957
SBP, mmHg	131 ± 16	131 ± 18	0.888	129 ± 16	128 ± 16	0.173
DBP, mmHg	86 ± 11	86 ± 12	0.747	84 ± 13	84 ± 14	0.288
CHD, n (%)	0 (0)	0 (0)	–	30 (2.6)	16 (3.9)	0.281
CBD, n (%)	0 (0)	0 (0)	–	24 (3)	17 (3)	0.180
Hypertension, n (%)	102 (28.0)	34 (25.0)	0.617	225 (19.4)	94 (22.8)	0.140
Dyslipidemia, n (%)	112 (30.8)	28 (21.2)	0.622	718 (61.9)	226 (54.9)	0.164
MetS, n (%)	128 (56.1)	58 (31.2)	0.074	663 (57.2)	234 (56.8)	0.886
DR, n (%)	–	–	–	332 (28.6)	113 (27.4)	0.797
DKD, n (%)	–	–	–	169 (14.6)	77 (18.7)	0.049
FPG, mmol/l	5.4 ± 0.4	5.4 ± 0.4	0.787	8.7 ± 2.4	8.8 ± 2.4	0.231
FINS, μU/ml	6.9 (4.8,9.8)	7.8 (4.9,11.2)	0.068	8.6 (5.8,13.5)	9.0 (5.8,13.7)	0.635
HbA1c, %	5.4 ± 0.3	5.4 ± 0.3	0.548	8.1 ± 1.9	8.2 ± 1.9	0.203
TC, mmol/l	4.9 ± 0.8	4.9 ± 0.8	0.954	5.0 ± 1.3	4.8 ± 1.1	0.001
HDL, mmol/l						
Male	1.1 ± 0.3	1.1 ± 0.2	0.5	1.0 ± 0.3	1.0 ± 0.3	0.364
Female	1.2 ± 0.2	1.2 ± 0.3	0.498	1.2 ± 0.3	1.28 ± 0.3	0.79
LDL, mmol/l	2.9 ± 0.7	2.9 ± 0.7	0.511	3.0 ± 0.9	2.9 ± 0.9	0.147
TG, mmol/l	1.0 (0.7,1.6)	1.1 (0.8,1.6)	0.221	1.8 (1.3,2.6)	1.7 (1.2,2.4)	0.119
UA, μmol/l						
Male	304.6 ± 73.8	312.4 ± 65.4	0.430	345.1 ± 91.8	353.8 ± 84.8	0.333
Female	231.9 ± 56.0	231.1 ± 51.2	0.925	290.4 ± 81.6	309.1 ± 85.4	0.07
ALT, U/l	18.0 (14.0, 24.0)	19.0 (15.0, 25.8)	0.178	22.0 (16.0, 35.0)	22.0 (15.8, 35.0)	0.570
AST, U/l	20.0 (18.0, 24.0)	21.0 (18.0, 25.0)	0.979	21.0 (17.0, 27.0)	20.0 (16.0, 28.0)	0.422
CRE, µmol/l	62.0 ± 32.0	61.7 ± 14.1	0.911	65.9 ± 19.0	65.3 ± 18.4	0.658
eGFR, ml/min*1.73 m^2^	129.3 ± 32.1	129.2 ± 29.7	0.961	125.1 ± 37.6	126.7 ± 37.1	0.598
ACR, mg/g	5.1 (1.8,12.0)	4.8 (2.1,11.4)	0.992	10.0 (3.5,47.9)	13.3 (4.3,54.4)	0.214
HOMA-IR	1.6 (1.1,2.4)	1.9 (1.2,2.8)	0.084	3.3 (2.1,5.0)	3.3 (2.1,5.4)	0.254
HOMA-β	75.4 (52.7,110.2)	89.3 (53.0,117.9)	0.107	37.6 (21.7,67.4)	37.4 (21.1,66.7)	0.643

NGT, normal glucose tolerance; DM, diabetes mellitus; BMI, body mass index; WC, waist circumference; SBP, systolic blood pressure; DBP, diastolic blood pressure; CHD, coronary heart disease; CBD, cerebrovascular disease; DR, diabetic retinopathy; DKD, diabetic kidney disease; FPG, fasting plasma glucose; FINS, fasting insulin; HbA1c, hemoglobin A1c; TC, total cholesterol; HDL-C, high-density lipoprotein cholesterol; LDL-C, low-density lipoprotein cholesterol; TG, triglycerides; UA, uric acid; eGFR, estimated glomerular filtration rate; CRE, creatinine; ACR, albumin-to-creatinine ratio; HOMA-IR, homeostasis model assessment estimated insulin resistance; HOMA-β, homeostasis model assessment β function.

Values are means ± SDs or numbers of subjects (percentages), and medians (interquartile range) were for non-normally distributed data. Student’s t-tests and chi-square tests were performed to compare the clinical difference between two groups. The Mann–Whitney rank-sum test was used to make comparisons for the non-normally distributed variable.

We divided the patients into two groups: (1) without variants and (2) with one-to-three variants. In the logistic regression analysis, compared with the group without the variants, the OR of DKD for the group with one-to-three variants was 1.422 (95% CI: 1.055, 1.915, *P* = 0.021). After adjustments for age, the OR was 1.434 (95% CI: 1.061, 1.937, *P* = 0.019). In the adjusted model 3 that was further fitted with age and sex, the OR of DKD for the group with one-to-three variants was 1.433 (95% CI: 1.060, 1.936, P = 0.019).

The 20 mtDNA variants were in six domains of mitochondria including complex I, complex III, complex V, non-coding, rRNA, and rRNA. There was no significant difference of the prevalence of patients with normal BMI/overweight/obesity, FINS tertiles, HOMA-IR tertiles, and HOMA-β tertiles between patients with variants in these domains of mitochondria and without these variants in **Supplementary Table 3** and **Table 4**.

## 4 Discussion

In our study, we analyzed whole mitochondrial DNA sequence variants in EOD with MID. We identified that seven variants, of which five variants were in complex I of mitochondrial, one variant in complex III, and one in tRNA from cohort 2 and cohort 3, were possibly diabetes associated. Patients with variants in the non-coding region had a higher percentage of obesity and levels of FINS. The patients with 20 homozygous variants had a significantly higher prevalence of DKD than those without these variants in diabetic patients. As we know, it was the first study to investigate the whole mtDNA sequence variants with diabetes and DKD in the Chinese population, and our study has provided a new insight into the mitochondrial genetic role in diabetes and its complication *via*.

Mitochondria are essential in energy metabolism and cellular survival and play a central role in numerous metabolic processes. IR plays a key role in the development of T2DM and DKD. Several abnormalities in mitochondrial oxidative metabolism, including the reduced content and size of mitochondria (11), impaired mitochondrial respiration, and reduced electron transport chain activity (11, 12, 17), as well as the transcriptional downregulation of genes involved in mitochondrial oxidative metabolism (13, 14, 19), have been associated with obesity, T2DM, and aging in human skeletal muscle. These alterations are collectively termed mitochondrial dysfunction and are often associated with an impaired insulin-mediated increase in transcript levels and ATP synthesis in the mitochondria of skeletal muscle in insulin-resistant individuals (9, 38). Skeletal muscle is essential for metabolism because of its role in glucose uptake and its importance in exercise. It plays an important role in peripheral IR (71). In our study of cohort 2, we found that 42 rare variants were associated with IR. In addition, patients with variants in the non-coding region had a higher percentage of obesity and higher FINS. These variants were in the D-loop region, and previously, variants in the D-loop have been shown to be associated with breast cancer and other tumors (22, 68–70). The D-loop is the main regulation region of mitochondrial transcription (72). In addition, variants in the 12S rRNA gene were found to have a higher percentage of obesity. These variants in the 12S rRNA gene have been reported to be associated with a coronary atherosclerosis risk, deafness, and ischemic stroke (24–28). These data strongly indicate that the D-loop region and 12S rRNA may be associated with obesity. After enlarging the sample size, we found that there was an association of mitochondrial variants with DKD. The levels of FINS and HOMA-IR had an upward trend in the group with 20 variants, although there was no significant statistical difference. This may be related to the heteroplasmy of mitochondria and the loss of information by high-throughput sequencing.

Mitochondrial respiratory Complex I plays an important role in the process of ATP synthesis, which is the rate-limiting enzyme in electron transport chain (ETC) (73, 74). Current evidence suggests that the defects in complex I may be associated with metabolic diseases, including diabetes, IR, and NAFLD (3–5). In addition, complex I is also a major source of reactive oxygen species (ROS) in mitochondria (75), which has been implicated in the pathology of Parkinson disease (76) and ageing (77). Previous studies have demonstrated that the pivotal role of the ND6 epigenetic network could regulate mitochondrial function and affect IR (5). It indicated that the function of complex I may be associated with IR. Five of the above seven mtDNA variants were found in complex I in our study, which were previously reported in patients with diabetes (34, 38, 40, 78). It may be due to the influence on the structure and the function of complex I, leading to IR and defecting the function of islets; thereby, these were considered to be possibly diabetes and DKD associated.

m.A10005G was previously reported to be possibly associated with hearing loss (45). m.A10005G is located in the D-loop and had a crucial role in tRNA recognition by cognate aminoacyl-tRNA synthetases (79). Another variant was the m.A15218G variant in MT-CYB. Previous research associated m.A15218G with epilepsy, diabetes, and Leber hereditary optic neuropathy (LHON) (78, 80). This variant led to the substitution of a conserved hydrophilic threonine by a hydrophobic alanine at position 159 of the cytochrome b subunit of complex III. In addition, the position was highly conservative (81). The cytochrome b subunit of complex III formed the catalytic core (82). The evolutionary conservation of the amino acid position, results from the prediction algorithms, and the homoplasic nature of the variant suggest that m.A15218G is mildly deleterious (64).

Current studies have shown that increased ROS was associated with diabetic kidney injury (83). Hyperglycemia would stimulate an increase in aerobic glycolysis with the resultant increase in substrate delivery to the mitochondria and a subsequent increase in mitochondrial activity (84). Increased tricarboxylic acid cycle (TCA) activity would produce more NADH and FADH2, which would then be used in the ETC in the mitochondria. The above evidence may partly explain the reason why the mtDNA variation could lead to DKD. The variety of mtDNA variants could alternate the structure of mitochondria and affect different aspects of mitochondrial function, thereby causing diabetes and DKD.

Diabetes due to mtDNA variants requires individualized treatment. Metformin is not recommended in the case of mitochondrial diabetes because it was shown to cause lactic acidosis. SGLT-2i could lead to lower blood sugar and body weight; reduced cardiovascular events risks; and the improvement of the inflammation status, liver steatosis, and uric acid concentrations (85). GLP-1 Ras also had the protective effect on the cardiovascular system. Since mtDNA variants are known to elevate the level of ROS, they are especially dangerous for the cardiovascular system, where they can cause cardiomyopathy, atherosclerosis, and hypertension. Diabetic patients with identified mtDNA variants are considered a high-risk group for cardiovascular diseases. Therefore, the cardioprotective effects of SGLT-2i, GLP-1 RA, and related substances have significant benefits in comparison to alternative treatments (86). In addition, we also need to pay attention to new strategies aimed at restoring complex I activity, reducing oxidative stress in alleviating IR, and the treatment of diabetes.

There are also three limitations in our study. First, the software we used was aimed to evaluate the coding variants and tRNA, and the variants in rRNA were unavailable. The pathogenicity of these variants is needed to be further investigated. Secondly, the sample size to evaluate the potential disease-associated variants was limited; thus, a larger sample size of population is needed to further investigate the variants in the mitochondrial diabetes and DKD for future studies. Thirdly, the exact mechanism of the potential disease-associated variants that possibly cause disease is further needed for the research *in vivo* and *in vitro* studies.

## 5 Conclusions

In our study, we found that seven variants in mitochondrial complex I, D-loop, and complex I were possibly associated with diabetes and 20 mtDNA variants may be associated with a higher risk of DKD. Although it has been proposed that the variants might impair the function of mitochondrial complex I and elevate the levels of ROS, the molecular mechanism linking diabetes and DKD is further needed to be investigated. Prospective and larger-scale studies are needed to establish the clinical relevance of this association.

## Data availability statement

The datasets presented in this article are not readily available due to ethical restrictions. Requests to access the datasets should be directed to the corresponding author.

## Ethics statement

The studies involving human participants were approved by the Ethics Committee of Peking University People’s Hospital. The patients/participants provided their written informed consent to participate in this study.

## Author contributions

ML conducted this study, collected and explored the data, and completed the original manuscript. LJ contributed to the study design, critically revised the manuscript, and obtained the funding. XH also contributed to the study design and critically revised the manuscript. SG contributed to the sample collection and analysis of the data and reviewed the manuscript. LZ, SZ, QR, XC, YYL, WL, YZ, XZ, and YFL contributed to the data and sample collection and reviewed the manuscript. All authors read the final manuscript and approved final submission.

## Funding

This research was supported by grants from National Key Research and Development Program of China (2016YFC1304901), Beijing Municipal Science and Technology Committee Funding (Z141100007414002 and D131100005313008), National High-Technology Research and Development Program of China (2012AA02A509), Bethune Merck Research Fund for young and middle-aged doctors (2119000314), and Major Chronic Non-communicable Disease Prevention and Control Research(2016YFC1305600).

## Acknowledgments

We would like to express our gratitude to the subjects for their contributions to the study, as well as to all the physicians and nurses for their work in this procedure of the study.

## Conflict of interest

The authors declare that the research was conducted in the absence of any commercial or financial relationships that could be construed as a potential conflict of interest.

## Publisher’s note

All claims expressed in this article are solely those of the authors and do not necessarily represent those of their affiliated organizations, or those of the publisher, the editors and the reviewers. Any product that may be evaluated in this article, or claim that may be made by its manufacturer, is not guaranteed or endorsed by the publisher.

## Abbreviations

mtDNA, mitochondrial DNA; NGS, next-generation sequencing; EOD, early-onset diabetes; MID, maternally inherited diabetes; DKD, diabetic kidney disease; tRNA, transfer RNA; IR, insulin resistance; NAFLD, non-alcoholic fatty liver disease; MetS, metabolic syndrome; FINS, fasting insulin; FPG, fasting plasma glucose; HOMA, homeostasis model assessment; HbA1c, hemoglobin A1c; LHON, Leber hereditary optic neuropathy.
