# Supplementary material for: Contribution of mitochondrial gene variants in diabetes and diabetic kidney disease
Source: Front Endocrinol (Lausanne). 2022 Oct 12;13:953631. doi: 10.3389/fendo.2022.953631 (PMC9597463; doi:10.3389/fendo.2022.953631)
Supplement: Supplementary file 1 [file DataSheet_1.pdf]

Supplementary Table 1 PCR primers used for mtDNA amplification

Primers	Forward primer	Reverse primer	Length
1F	CTCCTCAAAGCAATACACTG	TGCTAAATCCACCTTCGACC	840
2F	CGATCAACCTCACCACCTCT	TGGACAACCAGCTATCACCA	802
3F	GGACTAACCCCTATACCTTCTGC	GGCAGGTCAATTTCACTGGT	860
4F	AAATCTTACCCCGCCTGTTT	AGGAATTGCCATTGCGATTAG	887
5F	TACTTCACAAAGCGCCTTCC	ATGAAGAATAGGGCGAAGGG	832
6F	TGGCTCCTTTAACCTCTCCA	AAGGATTATGGATGCGGTTG	898
7F	ACTAATTAATCCCCTGGCCC	CCTGGGGTGGGTTTTGTATG	975
8F	CTAACCGGCTTTTTGCCC	ACCTAGAAGGTTGCCTGGCT	814
9F	GAGGCCTAACCCCTGTCTTT	ATTCCGAAGCCTGGTAGGAT	827
10F	CTCTTCGTCTGATCCGTCCT	AGCGAAGGCTTCTCAAATCA	886
11F	ACGCCAAAATCCATTTCACT	CGGGAATTGCATCTGTTTTT	987
12F	ACGAGTACACCGACTACGGC	TGGGTGGTTGGTGTAAATGA	900
13F	TTTCCCCCTCTATTGATCCC	GTGGCCTTGGTATGTGCTTT	816
14F	CCCACCAATCACATGCCTAT	TGTAGCCGTTGAGTTGTGGT	940
15F	TCTCCATCTATTGATGAGGGTCT	AATTAGGCTGTGGGTGGTTG	891
16F	GCCATACTAGTCTTTGCCGC	TTGAGAATGAGTGTGAGGCG	840
17F	TCACTCTCACTGCCCAAGAA	GGAGAATGGGGGATAGGTGT	802
18F	TATCACTCTCCTACTTACAG	AGAAGGTTATAATTCCTACG	866
19F	AAACAACCCAGCTCTCCCTAA	TCGATGATGTGGTCTTTGGA	977
20F	ACATCTGTACCCACGCCTTC	AGAGGGGTCAGGGTTCATTC	970
21F	GCATAATTAAACTTTACTTC	AGAATATTGAGGCGCCATTG	938
22F	TGAAACTTCGGCTCACTCCT	AGCTTTGGGTGCTAATGGTG	1162
23F	TCATTGGACAAGTAGCATCC	GAGTGGTTAATAGGGTGATAG	765
24F	CACCATTCTCCGTGAAATCA	AGGCTAAGCGTTTTGAGCTG	954

Supplementary Table 2 Clinical features of patients carrying the m.A3943G and m.A10005G variants which were just in diabetic patients

Variant	Sex	Age at diagnosis (years)	Age (years)	BMI (kg/m^2)	HbA1c (%)	FPG (mmol/l)	FINS (uU/ml)	FCP (ng/ml)	ACR (mg/g)	eGFR	TG	LDL	DR
m.A3943G	M	34	38	30.8	10.5	9.0	11.71	1.71	24.35	138.61	1.13	3.34	Yes
m.A10005G	M	32	35	28.0	11.2	15.96	15.33	2.79	8.93	137.96	4.91	2.11	No

Abbreviations: M, Male. F, Female; BMI, body mass index (calculated as body mass in kilograms divided by height in meters, squared); HbA1c, haemoglobin A1c; FPG, fasting plasma glucose; FINS, fasting insulin; FCP, fasting C-peptide; ACR, albumin/creatinine ratio; DR, diabetic retinopathy.

Supplementary Table 3 Comparison of BMI and other traits among the patients with T2D from cohort 3 with variants and without these variants in different domains of mitochondria

	Tertile/Category	In complex I		P-value	In complex III		P- value	In complex V		P-value
		Without variants	With variants		Without variants	With variants		Without variants	With variants	
BMI	normal	426/1341 (31.8%)	66/210	0.964	476/1505 (31.6%)	16/46 (34.8%)	0.854	460/1435 (32.1%)	32/116 (27.6%)	0.172
	overweight	613/1341 (45.7%)	98/210		690/1505 (45.8%)	21/46 (45.7%)		661/1435 (46.1%)	50/116 (43.1%)	
	obesity	302/1341 (22.5%)	46/210		339/1505 (22.5%)	9/46 (19.6%)		314/1435 (21.9%)	34/116 (29.3%)	
FINS*	T1	336/1148 (29.3%)	61/182 (33.5%)	0.502	386/1293 (29.9%)	11/37 (29.7%)	0.712	369/1227 (30.1%)	28/103 (27.2%)	0.811
	T2	376/1148 (32.8%)	57/182 (31.3%)		423/1293 (32.7%)	10/37 (27.0%)		399/1227 (32.5%)	34/103 (33.0%)	
	T3	436/1148 (38.0%)	64/182 (35.2%)		484/1293 (37.4%)	16/37 (43.2%)		459/1227 (37.4%)	41/103 (39.8%)	
HOMA-IR*	T1	259/1138 (22.8%)	47/181 (26.0%)	0.427	298/1284 (23.2%)	8/35 (22.9%)	0.997	288/1216 (23.7%)	18/103 (17.5%)	0.294
	T2	391/1138 (87.9%)	54/181 (29.8%)		433/1284 (33.7%)	12/35 (34.3%)		405/1216 (33.3%)	40/103 (38.8%)	
	T3	488/1138 (42.9%)	80/181 (44.2%)		553/1284 (43.1%)	15/35 (42.9%)		523/1216 (43.0%)	45/103 (43.7%)	
HOMA-β*	T1	488/1139 (42.8%)	81/181 (44.8%)	0.844	553/1284 (43.1%)	16/36 (44.4%)	0.784	528/1217 (43.4%)	41/103 (39.8%)	0.635
	T2	376/1139 (33.0%)	56/181 (30.9%)		419/1284 (32.6%)	13/36 (36.1%)		394/1217 (32.4%)	38/103 (36.9%)	
	T3	275/1139 (24.1%)	44/181 (24.3%)		312/1284 (24.3%)	7/36 (19.4%)		295/1217 (24.2%)	24/103 (23.3%)	

Abbreviations: BMI, body mass index; FINS, fasting insulin; HOMA-IR, homeostasis model assessment insulin resistance index; HOMA-β, homeostasis model assessment β-cell function; T, tertile.

Notes: T1-3 were grouped according to tertile category of FINS, HOMA-IR. Ordered classification chi square test was used to compare the differences between groups. When we analyzed the difference of FINS and HOMA-IR, patients using insulin were excluded. * means that subjects who were treated with insulin were excluded. P-value <0.05 was considered significant.

Supplementary Table 4 Comparison of BMI and other traits among the patients with T2D from cohort 3 with variants and without these variants in different domains of mitochondria

	Tertile/Category	In non-coding		P-value	In rRNA		P-value	In tRNA		P-value
		Without variants	With variants		Without variants	With variants		Without variants	With variants	
BMI	normal	485/1521 (31.9%)	7/30 (23.3%)	0.595	468/1498 (31.2%)	24/53 (45.3%)	0.096	474/1512 (31.3%)	18/39 (46.2%)	0.146
	overweight	696/1521 (45.8%)	15/30 (50.0%)		691/1498 (46.1%)	20/53 (37.7%)		697/1512 (46.1%)	14/39 (35.9%)	
	obesity	340/1521 (22.4%)	8/30 (26.7%)		339/1498 (22.6%)	9/53 (17.0%)		341/1512 (22.6%)	7/39 (17.9%)	
FINS*	T1	391/1306 (29.9%)	6/24 (25.0%)	0.444	384/1283 (29.9%)	13/47 (27.7%)	0.943	387/1294 (29.9%)	10/36 (27.8%)	0.446
	T2	427/1306 (32.7%)	6/24 (25.0%)		417/1283 (32.5%)	16/47 (34.0%)		424/1294 (32.8%)	9/36 (25.0%)	
	T3	488/1306 (37.4%)	12/24 (50.0%)		482/1283 (37.6%)	18/47 (38.3%)		483/1294 (37.3%)	17/36 (47.2%)	
HOMA-IR*	T1	302/1296 (23.3%)	4/23 (17.4%)	0.648	297/1272 (23.3%)	9 (2.9%)	0.795	296/1283 (23.1%)	10/36 (27.8%)	0.517
	T2	438/1296 (33.8%)	7/23 (30.4%)		428/1272 (33.6%)	17 (3.8%)		436/1283 (34.0%)	9/36 (25.0%)	
	T3	556/1296 (42.9%)	12/23 (52.2%)		547/1272 (43.0%)	21 (3.7%)		551/1283 (42.9%)	17/36 (47.2%)	
HOMA-β*	T1	562/1297 (43.3%)	7/23 (30.4%)	0.278	553/1273 (43.4%)	17/47 (36.2%)	0.593	551/1284 (42.9%)	18/36 (50.0%)	0.579
	T2	421/1297 (32.5%)	11/23 (47.8%)		414/1273 (32.5%)	18/47 (38.3%)		423/1284 (32.9%)	9/36 (25.0%)	
	T3	314/1297 (24.2%)	5/23 (21.7%)		307/1273 (24.1%)	12/47 (25.5%)		310/1284 (24.1%)	9/36 (25.0%)	

Abbreviations: BMI, body mass index; FINS, fasting insulin; HOMA-IR, homeostasis model assessment insulin resistance index; HOMA-β, homeostasis model assessment β-cell function; T, tertile.

Notes: T1-3 were grouped according to tertile category of FINS, HOMA-IR. Ordered classification chi square test was used to compare the differences between groups. When we analyzed the difference of FINS and HOMA-IR, patients using insulin were excluded. * means that subjects who were treated with insulin were excluded. P-value <0.05 was considered significant.
